# Inhibition of Bacterial and Fungal Biofilm Formation by 675 Extracts from Microalgae and Cyanobacteria

**DOI:** 10.3390/antibiotics8020077

**Published:** 2019-06-12

**Authors:** Virginio Cepas, Yuly López, Yaiza Gabasa, Clara B. Martins, Joana D. Ferreira, Maria J. Correia, Lília M.A. Santos, Flávio Oliveira, Vitor Ramos, Mariana Reis, Raquel Castelo-Branco, João Morais, Vitor Vasconcelos, Ian Probert, Emilie Guilloud, Mohamed Mehiri, Sara M. Soto

**Affiliations:** 1 ISGlobal, Hospital Clínic—Universitat de Barcelona, 08036 Barcelona, Spain; virginio.cepas@isglobal.org (V.C.); yuly.lopez@isglobal.org (Y.L.); gabasagyaiza@gmail.com (Y.G.); 2 Coimbra Collection of Algae (ACOI), Department of Life Sciences, University of Coimbra, 3000-456 Coimbra, Portugal; martinscsb@gmail.com (C.B.M.); ferreirajoanadias@gmail.com (J.D.F.); mariajacorreia1@gmail.com (M.J.C.); liliamas@ci.uc.pt (L.M.A.S.); 3 Interdisciplinary Centre of Marine and Environmental Research (CIIMAR), 4450-208 Porto, Portugal; up201510053@fc.up.pt (F.O.); vtr.rms@gmail.com (V.R.); mariana.a.reis@gmail.com (M.R.); raquel.castelobranco.12@gmail.com (R.C.-B.); joaopmorais@gmail.com (J.M.); vmvascon@fc.up.pt (V.V.); 4 Faculty of Sciences, University of Porto, 4450-208 Porto, Portugal; 5 Roscoff Culture Collection, Sorbonne University/CNRS, Roscoff Biological Station, 29680 Roscoff, France; probert@sb-roscoff.fr (I.P.); emilie.guilloud@gmail.com (E.G.); 6 Marine Natural Products Team, Nice Institute of Chemistry, UMR 7272 University Nice Côte d’Azur/CNRS, 60103 Nice, France; Mohamed.MEHIRI@unice.fr

**Keywords:** Marine sources, Microalgae, Cyanobacteria, Antibiofilm

## Abstract

Bacterial biofilms are complex biological systems that are difficult to eradicate at a medical, industrial, or environmental level. Biofilms confer bacteria protection against external factors and antimicrobial treatments. Taking into account that about 80% of human infections are caused by bacterial biofilms, the eradication of these structures is a great priority. Biofilms are resistant to old-generation antibiotics, which has led to the search for new antimicrobials from different sources, including deep oceans/seas. In this study, 675 extracts obtained from 225 cyanobacteria and microalgae species (11 phyla and 6 samples belonging to unknown group) were obtained from different culture collections: The Blue Biotechnology and Ecotoxicology Culture Collection (LEGE-CC), the Coimbra Collection of Algae (ACOI) from Portugal, and the Roscoff Culture Collection (RCC) from France. The largest number of samples was made up of the microalgae phylum *Chlorophyta* (270) followed by Cyanobacteria (261). To obtain a large range of new bioactive compounds, a method involving three consecutive extractions (hexane, ethyl acetate, and methanol) was used. The antibiofilm activity of extracts was determined against seven different bacterial species and two *Candida* strains in terms of minimal biofilm inhibitory concentration (MBIC). The highest biofilm inhibition rates (%) were achieved against *Candida albicans* and *Enterobacter cloacae*. *Charophyta*, *Chlorophyta*, and Cyanobacteria were the most effective against all microorganisms. In particular, extracts of *Cercozoa* phylum presented the lowest MBIC_50_ and MBIC_90_ values for all the strains except *C. albicans*.

## 1. Introduction

Bacterial biofilms are formed by aggregates of microorganisms within a complex biological system composed of assemblages of sessile cells adherent to each other or to a surface. Biofilms are embedded in an extracellular polymeric substance (EPS) matrix composed mainly of polysaccharides, lipids, proteins, and external DNA (eDNA) [1]. Biofilms provide bacteria protection against external factors such as temperature, pH variations, desiccation, oxidization, ultraviolet radiation, and metal ions. Furthermore, biofilms are able to evade innate and/or adaptive immune defenses and avoid antimicrobial treatments by several mechanisms [2,3,4]. Biofilms have the capacity to attach to both biotic and abiotic surfaces, increasing the colonization of medical devices such as urinary and intravenous catheters, mechanical heart valves, endotracheal tubes, and prosthesis joints [5]. In fact, the National Institute of Health (NIH—United States of America) recognizes that 80% of human infections are caused by bacterial biofilms [6,7]. In the United States, biofilm-related infections affect over 12 million people with an estimated annual economic burden of 6 billion dollars [8]. 

On the other hand, in the last several decades, antimicrobial resistance (AMR) has increased becoming a serious problem worldwide. Infections caused by drug-resistant bacteria increase the risk of death compared with non-resistant bacteria. However, the emergence of antimicrobial resistant microorganisms has been accompanied by a decrease in the number of new antibacterial agents in the market. Indeed, only two new classes of anti-bacterial drugs have been marketed in the last 60 years: linezolid representing the oxazolidinone group, and daptomycin representing the lipopeptide group [9]. The problem of AMR is further enhanced when current treatments cannot completely eradicate persistent cells that remain inside the biofilm. There is a well-established correlation between bacterial persistence and microorganisms able to form biofilm [10].

In addition, apart from bacterial infections rising in hospitals, the incidences of fungal infections are growing with the development of resistance to conventional antifungal agents [11]. The complex three-dimensional structures of biofilms form a favorable environment for micro niches of Candida species. The biofilm occurrence of these organisms contributing to numerous infections [12]. The fungal pathogen most commonly associated with biofilm infections is *Candida albicans*, these infections being associated with a high mortality. The most common sites for fungal infections associated with biofilms are the oral cavity, lungs, burn wounds, the lower reproductive tract, the gastrointestinal tract, skin, intravascular and the insertion site of urinary catheters [13]. Thus, *C. albicans* and *C. parapsilosis* are two of the Candida species more frequently associated with symptomatic vulvovaginal candidiasis, biofilm formation being essential for the development of this type of infection [14]. These structures confer Candida species a high resistance to the antifungal used in their treatment.

Therefore, the importance of finding new bioactive compounds arises from global bacterial resistance to existing antibiotics. One of the possible sources of new antibiofilm and antimicrobial agents are marine organisms such as macroalgae, microalgae, bryozoans, cnidarians, echinoderms, sponges, molluscs, tunicates, marine fungi, and marine bacteria [15]. Indeed, in 2015, 1340 new marine natural products (MNPs) were reported to have potential efficacy against cancer, viruses, bacteria, fungi, hypertension, high cholesterol, and other diseases [16]. Nevertheless, antimicrobial activity has been detected in 262 marine compounds including alkaloids, terpenoids, lipids, peptides, halogenated compounds, polyketides, isocumarins, nucleosides, and other minority compounds found in MNPs [17]. Several studies have described the antimicrobial activity of a very diverse array of MNPs from marine species [16,18]. In particular, microalgae derivatives may be potentially promising candidates for the development of novel antibacterial drugs because of their ability to combat pathogenic bacteria found throughout the ocean [19,20]. These microorganisms have been described as rich sources of several bioactive compounds such as proteins, fatty acids, vitamins, and pigments [20]. Additionally, the coexistence of several species in aquatic systems creates a competitive niche that can lead them to release compounds into the environment in order to facilitate advantage over competitors [21]. These compounds have shown antifungal, antiviral, antialgal, antienzymatic, or antibiotic activity [20]. Lipids such as short-chain fatty acids and PUFAs (polyunsaturated fatty acids) have been associated with antibacterial properties [22,23]. Nonetheless, there is a lack of data related to antibiofilm activity of MNPs obtained from microalgae and cyanobacteria species, with the exception of a single brief reference [24]. 

The aim of the present study was to determine the antibiofilm activity of microalgae and cyanobacteria species against nine biofilm-forming human pathogens, representing the most important Gram-positive, Gram-negative, and fungal species, to search for new bioactive antibiofilm compounds using the biofilm inhibition ratio (%) and minimal biofilm inhibitory concentration (MBIC) assay. 

## 2. Results

The results showed that 205 hexane extracts exhibited the best antibiofilm activity, followed by 195 extracts obtained with methanol and 189 extracts presenting inhibitory activity obtained with ethyl acetate. The rest of the extracts did not show any antibiofilm activity. Nevertheless, no significant activity was reported between methods of extraction (*p* > 0.05) (Table S1). The small differences in activity among the three solvents suggested that the three solvent protocol covers a large range of compounds with different polarities, and it is more effective than extraction with only one solvent.

**Figure 1:**
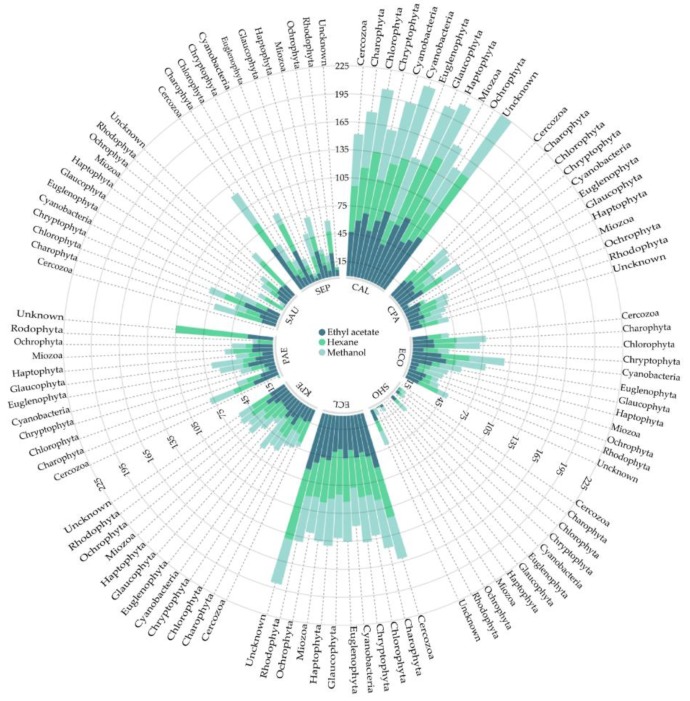
Circular dot plot representing the biofilm inhibition ratio (%) of each bacterium in relation to the solvent employed (ethyl acetate, hexane, and methanol), according to the microalgae and cyanobacteria phylum. CAL: *C. albicans*; CPA: *C. parapsilopsis*; ECO: *E. coli*; SHO: *S. hominis*; ECL: *E. cloacae*; KPE: *K. pneumoniae*; PAE: *P. aeruginosa*; SAU: *S. aureus*; SEP: *S. epidermidis*.

**Figure 2:**
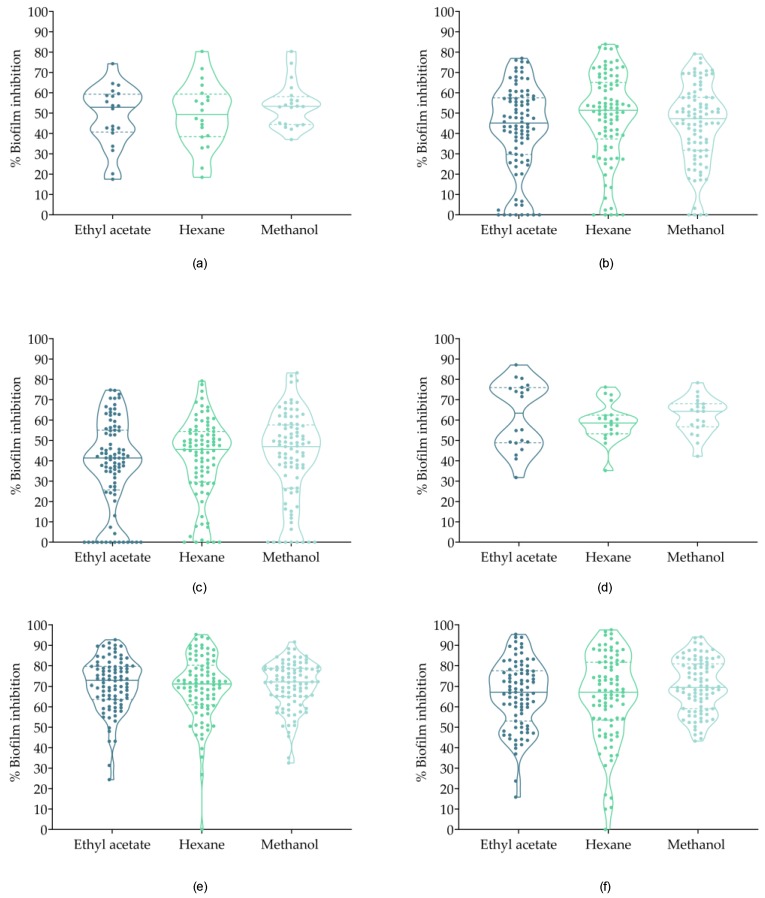
Individual biofilm inhibition ratios of *Charophyta*, *Chlorophyta*, and Cyanobacteria extracts against *E. cloacae* and *C. parapsilopsis*, represented as percentages. (**a**): *Charophyta* against *E. cloacae*; (**b**) *Chlorophyta* against *E. cloacae*; (**c**) Cyanobacteria against *E. cloacae*; (**d**) *Charophyta* against *C. albicans*; (**e**) *Chlorophyta* against *C. albicans*; (**f**) *Cyanobacteria* against *C. albicans*.

Figure 1 provides an overview of the biofilm inhibition ratio (%) per group and solvent. The highest inhibition ratios were reported in *C. albicans* and *E. cloacae* in all solvents. Interestingly, *C. albicans* showed high inhibition rates above 50% of inhibition in all samples, with the exception of *Glaucophyta* and *Miozoa* methanol extracts (28.2% and 12.55%, respectively) and *Rhodophyta* hexane extract (34.77%). High biofilm inhibition ratios, about 35%, were found for *E. cloacae*. These rates were lower compared to *C. albicans* but still, more active in comparison with the rest of microorganisms. In the case of *E. cloacae*, only the methanol extract from the *Miozoa* phylum showed activity below 35% (9.38%). Biofilm inhibition of the nine microorganisms tested was individually analyzed in Figure 2. The inhibition rates in both cases were remarkably high and almost 50% of inhibitions were above the median value. On the other hand, in the case of *S. hominis*, only the methanol extract from *Miozoa* was able to inhibit it up to 64.22%. In addition, extract obtained from *Cercozoa* and *Euglenophyta* did not present activity against *S. aureus*.

Only the results from phyla with a high number of extracts tested (*Charophyta*, *Chlorophyta* and Cyanobacteria) were statistically analyzed. The two-way ANOVA showed that both solvents and phylum significantly influenced the biofilm formation rates (*p* < 0.001) compared to growth control. 

To further investigate which phylum and method of extraction (solvent) was more effective in biofilm inhibition, Tukey’s multiple comparison test was also performed. *Charophyta* presented the best inhibition ratios against *E. coli*, *P. aeruginosa*, *S. aureus*, *S. epidermidis*, and *C. parapsilopsis* (Tables S2–S4). Cyanobacteria induced biofilm inhibition versus *K. pneumoniae* and *S. hominis*. On the other hand, the three phyla presented the same effectiveness against both *C. albicans* and *E. cloacae* (Tables S2–S4). 

*C. albicans* and *C. parapsilopsis* were inhibited by 308 extracts. Biofilm formation among Gram-negative strains was inhibited by 202 extracts (30%) with equal distribution among species. Among Gram-positive strains, biofilm formation was inhibited by 69 of the 675 samples (10.2%).

*Cercozoa* and the unknown group showed activity at the lowest concentration in all the groups of microorganisms, except for *C. albicans*. Among all microorganism, the *Cryptophyta*, *Euglenophyta*, and *Glaucophyta* groups showed the best antibiofilm activity. 

The Cercozoa presented an MBIC_50_ of 32 and an MBIC_90_ of 128 µg/mL in *E. coli*, *K. pneumoniae*, *E. cloacae*, *S. aureus*, and *C. parapsilopsis*, whereas among *S. epidermidis* and *S. hominis* the MBIC_50_ and MBIC_90_ were 64 and 256 µg/mL, respectively. *P. aeruginosa* showed the lowest inhibitory concentrations, with MBIC_50_ and MBIC_90_ values of 16 and 64 µg/mL, respectively. 

The unknown group (represented by two species) presented the highest activity in all microorganisms except for *E. cloacae*. Thus, the MBIC_50_ was 64 µg/mL, and the MBIC_90_ was 256 µg/mL for *E. coli*, *P. aeruginosa*, and *C. parapsilopsis. K. pneumoniae* and *S. hominis* showed MBIC_50_ and MBIC_90_ values of 64 and 128 µg/mL, respectively. Finally, *S. aureus* and *S. epidermidis* presented an MBIC_50_ of 128 µg/mL and an MBIC_90_ of 256 µg/mL. 

Among the Gram-negative bacteria (Table 1), *E. cloacae* was inhibited by the lowest MBIC values of all the extracts tested. In addition to the results described above, the *Cryptophyta* and *Rhodophyta* phyla demonstrated activity against *E. cloacae* with an MBIC_50_ and an MBIC_90_ ranging from 16 to 256 µg/mL and from 16 to 128 µg/mL, respectively. 

Individually, *Rhodophyta* showed antibiofilm activity against *E. coli* with MBIC_50_ and MBIC_90_ values of 64 and 512 µg/mL, respectively. 

The *Cryptophyta* showed activity against *P. aeruginosa* with MBIC_50_ and MBIC_90_ values of 64 and 512 µg/mL, respectively. 

Among Gram-positive bacteria (Table 2), *S. hominis* was inhibited by the lowest MBIC values of all the extracts studied. In addition to *Cercozoa* and the unknown group, the *Miozoa* showed MBIC_50_ and MBIC_90_ values of 128 and 1024 µg/mL, respectively. The activity of the *Miozoa* was only of note in *S. hominis*. Moreover, the *Haptophyta* demonstrated activity against *S. aureus* with MBIC_50_ and MBIC_90_ values of 128 and 512 µg/mL, respectively. Finally, *S. epidermidis* presented an MBIC_50_ of 256 µg/mL and an MBIC_90_ of 1024 µg/mL. 

Among *Candida* spp., the greatest activity was reported in *C. albicans* (Table 3). Three of the 11 groups were able to inhibit biofilm formation with MBIC_50_ values of 8 µg/mL. The lowest activity was reported by the *Euglenophyta* with an MBIC_50_ of 8 µg/mL and an MBIC_90_ of 16 µg/mL. Similar results were found with the *Cryptophyta* showing also an MBIC_50_ of 8 µg/mL and an MBIC_90_ of 128 µg/mL. Finally, the *Glaucophyta* presented an MBIC_50_ and an MBIC_90_ of 8 and 256 µg/mL, respectively. Interestingly, the *Euglenophyta* and *Glaucophyta* were only active against biofilm formation in *C. albicans*.

On the other hand, *Rhodophyta* species demonstrated activity against *C. parapsilopsis* with an MBIC_50_ of 64 µg/mL and an MBIC_90_ of 512 µg/mL.

## 3. Discussion

The present study was designed to determine the effect of new bioactive compounds from microalgae and cyanobacteria species on biofilm formation. We focused on these microorganisms because they are widely distributed in marine and freshwaters and are an important population in all strata, contributing as primary producers in open water systems [25,26,27,28]. Crude extracts are a heterogeneous mixture of polar and non-polar compounds. The selection of an efficient method of extraction is important for performing successive assays. For that reason, we used a method combining three different solvents (hexane [non-polar], ethyl acetate [polar], and methanol [polar]) for extracting a wide range of biological samples in order to collect the greatest range of polar and non-polar compounds. 

The purpose of hexane is to extract polar compounds from the mixture, such as triacylglycerides (TAG), while methanol and ethyl acetate, polar solvents, can extract many biological compounds (polar and non-polar metabolites), such as fatty acids (FA). 

In fact, previous studies have reported that FA have the ability to inhibit biofilm formation. For example, oleic acids block bacterial adhesion in *S. aureus* [29]. The *cis*-2-decenoic acid synthetized by *P. aeruginosa* is able to induce the dispersion of established biofilms and inhibit biofilm formation [30]. 

Indeed, recent studies have demonstrated that small FA messengers inhibit cell–cell communication, achieving biofilm dispersion and are considered to be a quorum sensing inhibitor [31]. Hence, with three consecutive extraction solvents we were able to collect a wide range of lipids including fatty acids (FA), waxes, sterols, hydrocarbons, ketones, and pigments (carotenoids, chlorophylls, and phycobilins) [32], which have been described as possessing high antibacterial and antibiofilm activity [23,33]. The findings of the one-way ANOVA data support the claim that three solvents are necessary to obtain a wide range of molecules. 

Only three phyla (*Charophyta*, *Chlorophyta*, and *Cyanobacteria*) were included in the two-way ANOVA analysis due to the number of samples extracted. Nevertheless, the fact that the other phyla showed interesting levels of biofilm inhibition rates (Figure 1) suggests that they are also good candidates for further studies.

The MBIC assay was used to determine the effectiveness of the sample extractions against biofilm producer microorganisms. Among bacteria, lower extract concentrations are needed to inhibit biofilm formation in Gram-negative in comparison with Gram-positive bacteria. Among the yeasts, lower extract concentrations are needed to inhibit biofilm formation in *C. albicans* compared to *C. parapsilopsis.* This finding was unexpected, and these extracts are being further investigated in an attempt to elucidate the mechanism underlying the antifungal action of these extracts.

The microalgae and cyanobacteria from which extracts generated were very diverse, and differences were observed in the level of bioactivity of different groups. 

It is interesting to note that extracts from the *Cercozoa* phylum were very active against the eight target microorganisms in this study, except *C. albicans*, which was relatively unaffected. The *Cercozoa* phylum is a very diverse lineage of unicellular amoeboid organisms that are mostly heterotrophic and that can have a very complex cellular ultrastructure and behavioral patterns. Common examples in marine environments include the biomineralizing radiolarians and Foraminifera. A single relatively minor group within this lineage, the chlorarachniophytes, has acquired green chloroplasts by secondary endosymbiosis at some point in its evolutionary history and therefore qualifies as microalgae. Very little is known about the ecology or metabolism of the 14 described species in this group, but members appear to be good candidates for further investigation in the context of the search for new bioactive molecules. However, these data should be interpreted with caution because the *Cercozoa* phylum was represented by only three marine culture strains in our study, all of which are relatively difficult to grow (low growth rates and maximum cell abundances, adherence to the surface of culture vessels), meaning scale-up of cultures is likely to be challenging. 

Several studies have described antimicrobial activities in microalgae and cyanobacteria species [34], but no results about antibiofilm against clinical pathogens, except those from Lauritano et al. [24], have been reported to date. Lauritano’s group found two species belonging to the *Leptocylindrus* genus showing strong antibiofilm activity against *S. epidermidis* when they are grown in N-starved medium.

This issue may be important since resistance against antimicrobial agents changes depending on the expression of the phenotype. Planktonic microorganisms show greater susceptibility against the current therapies available than biofilm-forming microorganisms [35,36]. On the other end of the bioactivity spectrum, the *Chlorophyta* and *Charophyta*, which are both lineages of green microalgae, consistently had high average MBIC values. The *Chlorophyta* are known for their ability to synthesize a variety of bioactive compounds such as lipids and derivative polyunsaturated fatty acids (PUFAs). Two PUFAs, docosahexaenoic acid (DHA) and eicosapentaenoic acid (EPA), have been demonstrated to exhibit antibacterial and antibiofilm properties [36,37]. Extracts from some chlorophyte strains had very low MBIC values and the high average values may be a reflection of the fact that the *Chlorophyta* was the most represented phylum in our study. Some microalgal groups appear to be specifically active against certain pathogens. For instance, extracts from *Rhodophyta* species exhibited relatively high antibiofilm activity against *E. cloacae*, while biofilm formation in *C. albicans* was particularly sensitive to extracts from *Cryptophyta*, *Euglenophyta*, and *Glaucophyta* (three completely unrelated lineages). Phyla such as Cyanobacteria, *Haptophyta* and *Ochrophyta* genera, all of which were fairly well represented in our study, consistently exhibited intermediate average MBIC values. These results can be explained due to several secondary metabolites such as circular or linear lipopeptides, amino acids, FA, macrolides, and amides with antibacterial activity [21].

## 4. Materials and Methods 

### 4.1. Microalgae and Cyanobacteria Extracts

A total of 225 species of microalgae and cyanobacteria (belonging to the phyla *Cercozoa*, *Charophyta*, *Chlorophyta*, *Cryptophyta*, Cyanobacteria, *Euglenophyta*, *Glaucophyta*, *Haptophyta*, *Miozoa*, *Ochrophyta*, *Rhodophyta*, and 2 unknown species) were tested. They were supplied by Blue Biotechnology and Ecotoxicology Culture Collection (LEGE-CC) at CIIMAR, Centro Interdisciplinar de Investigação Marinha e Ambiental (CIIMAR) [38], the Coimbra Collection of Algae, University of Coimbra (ACOI), and the Université Pierre et Marie Curie-Paris 6 (UPMC). ACOI microalgae strains were collected mainly from freshwater habitats in Portugal. Cyanobacteria were collected mainly from beaches along the Portuguese coast (Atlantic Ocean) but also from brackish and freshwater systems (rivers, lakes, and estuaries). Only one of the cyanobacteria strains tested in this study was isolated from Chile (lake).

Microalgae cultures were performed by CIIMAR, ACOI, and UPMC. Briefly, samples were cultured until stationary phase for 15 or 30 days, depending on the strain, with air bubbling, and temperature and pH ranged between 20 and 30 °C and between 6 and 8, respectively. 

The growth media used for microalgae culture were M7, BG-11 (Sigma-Aldrich C3061, Darmstadt, Germany), S2T2, and LC. Each culture was illuminated 14–16 h every day with fluorescent lamps at an intensity of 30–60 µmol/m2/s or 100–200 µmol/m2/s depending on the strain. 

Cyanobacteria strains were cultured for 30–45 days, depending on the strain, without bubbling, with daily manual shaking between 30 s and 1 min every day and temperature ranging between 25 and 30 °C. To culture cyanobacteria Z8 [39] and modified Z8 medium supplemented with 25 g/L of synthetic sea salts (Tropic Marin, Juliao do Tojal, Portugal) and B12 vitamin were used. The Z8 medium was supplemented because all marine cyanobacterial strains require artificial salts and some of them require the vitamin to grow. Cultures were performed under 14 h light /10 h dark cycles with a light intensity of 10–30 mol photons/m2/s).

Microalgae biomass was collected by centrifugation at 4000 rpm for 15 min. Pellets were frozen at −80 °C and lyophilized. Freeze-dried biomass was disrupted using a ceramic mortar, previously exposed to liquid nitrogen or with ultrasounds at 240 W, 35 kHz for 5 min.

Sequential extractions from hexane (non-polar), ethyl acetate to methanol (polar) were carried out. Each pellet was extracted by adding 3 × 20 mL of each solvent to centrifuge tubes, occasional vortexing and centrifuged at 4500 rpm for 15 min. Supernatant was recovered, transferred to glass vials, and fully dried in the rotary evaporator. Extracts were stored under dark dry conditions until use to avoid hydrolysis of bioactive molecules. The extracts were resuspended in 1 mL of 6% dimethyl sulfoxide (DMSO) (Panreac Applichem, Barcelona, Spain) immediately before the bioassay.

### 4.2. Microorganisms and Culture Conditions

Bacterial strains were stored in skim milk (BD) at −80 °C. In the present study, the strains were characterized in terms of biofilm formation, using the crystal violet protocol, as is shown in Table 4. *E. coli*, *K. pneumoniae*, *E. cloacae*, and *P. aeruginosa* were cultured for 24 h at 37 °C in aerobic conditions in Luria Broth agar (Condalab, Barcelona, Spain). 

*S. aureus*, *S. epidermidis*, and *S. hominis* were plated on Columbia agar with 5% sheep blood agar (Becton Dickinson, Huesca, Spain) for 24 h at 37 °C in aerobic conditions. 

*Candida* spp. strains were cultured for 24 h at 37 °C in Saboroud Agar (Becton Dickinson, Huesca, Spain). 

### 4.3. Minimal Biofilm Inhibitory Concentration (MBIC) 

The MBIC assay was performed by the broth microdilution assay described in the CLSI document M7-A7 with some modifications [40]. The culture media used for biofilm formation experiments was M63 supplemented with 0.25% glucose for *E. coli*, Luria Bertani broth supplemented with 0.25% glucose for *K. pneumoniae* and *P. aeruginosa*, Tryptic Soy Broth (TSB) supplemented with 0.25% glucose for Gram-positive bacteria, and Yeast Nitrogen Base (YNB) for *Candida* spp. These culture media improve the biofilm formation in the corresponding bacterial specie. 

For all strains, with an exception of *C. albicans*, two-fold dilution series of each microalgae extract in culture media was inoculated with 50 µL of a 0.5 McFarland Standard (corresponding to an inoculum of 5 × 10^6^ cells/well) and incubated for 48 h at 37 °C (or 30 °C in the case of *E. coli*) in aerobic conditions without shaking. A negative control (culture medium without inoculum) and a positive control (culture medium with inoculum) were included in each plate. All the plates were covered with adhesive foil lids to avoid evaporation. 

The biofilm susceptibility assay for *P. aeruginosa* was performed using the Calgary protocol as described previously [41] with one modification. The bacterial biofilm was formed by immersing the pegs of a modified polystyrene microtiter lid (catalog no. 445497; Nunc TSP system, Nunc, Roskilde, Denmark). 

For *Candida* spp., a loopful of yeasts, from overnight culture, were washed twice with 3 mL of phosphate-buffered saline (PBS; pH 7.2; Ca^2+^ and Mg^2+^-free), and the optical density of the suspension was adjusted to 0.38 at 520 nm. Two-fold dilution series of each microalgae extract in culture media was inoculated with 50 µL of a 0.38 OD (corresponding to an inoculum of 5 × 10^6^ cells/well) and incubated for 48 h at 37 °C in aerobic conditions without shaking.

For all microorganisms, after incubation, liquid culture was carefully removed and washed once with PBS and dried at 65 °C for at least 20 min. Biofilms were stained with 100 µL of 1% (*v/v*) solution of crystal violet (CV) and incubated for 10 min at room temperature. Afterwards, the CV was completely removed, washing once with PBS and heat-fixed at 65 °C for 60 min. 

The CV was eluted by the addition of 200 µL of 33% acetic acid. Biofilm formation was measured at 580 nm using a Microplate reader (EPOCH). The MBIC was defined as the lowest concentration of drug that resulted in a three-fold decrease of the optical density of 580 nm (OD_580_) in comparison with the positive growth-control value. The MBIC_50_ and MBIC_90_ values were calculated for all the strains.

### 4.4. Statical Analysis and Data Processing

The biofilm inhibition rates were calculated using the equation: 100 × (1 − OD_580_ of the test/OD_580_ of non-treated control). The MBIC_50_ and MBIC_90_ were defined as the lowest concentration that caused 50% and 90% inhibition on the formation of biofilm. Statistical analyses were performed in Prism 8 software (GraphPad Software, Inc., La Jolla, CA, USA). One-way ANOVA was used to compare the effects of solvents against bacterial biofilms. Two-way ANOVA with Tukey’s test was used to compare the phylum and solvents using OD values. Differences were considered statistically significant when *p* < 0.05. A circular dot plot was created with Tableau Software.

## 5. Conclusions

The present study provides initial large-scale evidence that microalgae and cyanobacteria are rich sources of substances with antibiofilm activity. Results demonstrated the importance of (1) employing a comprehensive extraction protocol in order to increase the chances of detecting bioactive substances; (2) testing extracts against a range of target microorganisms (because sensitivity to individual extracts differed among the organisms tested here); and (3) testing extracts from a broad range of source organisms (because significant differences were observed in the level of activity of extracts from different microalgal groups). Large-scale screening programs like this are extremely useful for identifying organisms that warrant further study as producers of bioactive substances of interest. Overall, the findings of this study provide insights for new opportunities provided by oceans and freshwater systems in the fight against biofilm infections. Further studies will be made in order to determine the active compounds responsible of the antibiofilm activity as well as their toxicity to mammal cells because at extract level toxicity could be due to other components different from the active one. 

## Figures and Tables

**Table 1:** Antibiofilm activity against Gram-negative bacteria of marine natural products. Range: range of concentrations tested; MBIC_50_: lowest concentration of the test compound that resulted in ≥50% inhibition of biofilm formation; MBIC_90_: lowest concentration of the test compound that resulted in ≥90% inhibition of biofilm formation; GM: geometric mean. MBICs are reported in µg/mL.

Phylum	*Escherichia coli*				*Klebsiella pneumoniae*				*Enterobacter cloacae*				*Pseudomonas aeruginosa*			
	Range	MBIC_50_	MBIC_90_	GM	Range	MBIC_50_	MBIC_90_	GM	Range	MBIC_50_	MBIC_90_	GM	Range	MBIC_50_	MBIC_90_	GM
*Cercozoa*	32–128	32	128	50.8	32–128	32	128	50.8	32–128	32	128	50.8	16–64	16	64	25.4
*Charophyta*	32–2048	256	512	82.5	32–2048	256	512	69.4	16–2048	256	512	145.3	64–2048	256	1024	326.3
*Chlorophyta*	8–4096	512	1024	301.7	8–4096	512	1024	315.2	2–4096	256	1024	288.1	8–4096	512	1024	315.2
*Cryptophyta*	16–1024	64	1024	101.6	8–1024	64	1024	90.5	4–256	16	256	22.6	8–512	64	512	64.0
Cyanobacteria	8–2048	128	1024	153.7	16–2048	256	1024	197.3	8–2048	256	512	186.6	8–2048	128	512	168.7
*Euglenophyta*	64–256	128	256	128.0	64–256	64	256	101.6	64–256	128	256	128.0	64–256	128	256	128.0
*Glaucophyta*	64–1024	256	1024	256.0	64–1024	256	1024	256.0	64–512	256	512	228.1	64–1024	256	1024	256.0
*Haptophyta*	16–512	128	512	136.3	16–512	128	512	128.0	4–512	64	512	72.6	8–512	64	256	82.3
*Miozoa*	32–1024	64	1024	122.2	32–1024	64	1024	116.7	16–1024	64	1024	101.6	32–1024	64	1024	111.4
*Ochrophyta*	32–1024	128	512	149.3	64–1024	128	512	157.2	8–512	64	512	91.7	32–1024	128	512	149.3
*Rhodophyta*	32–512	64	512	90.5	32–512	64	512	90.5	4–128	16	128	22.6	32–256	64	256	80.6
Unknown	16–256	64	256	71.8	16–128	64	128	64.0	16–128	64	128	64.0	16–256	64	256	71.8

**Table 2:** Antibiofilm activity against Gram-positive bacteria of marine natural products. Range: range of concentrations tested; MBIC_50_: lowest concentration of the test compound that resulted in ≥50% inhibition of biofilm formation; MBIC_90_: lowest concentration of the test compound that resulted in ≥90% inhibition of biofilm formation; GM: geometric mean. MBICs are reported in µg/mL.

Phylum	*Staphylococcus aureus*				*Staphylococcus epidermidis*				*Staphylococcus hominis*			
	Range	MBIC_50_	MBIC_90_	GM	Range	MBIC_50_	MBIC_90_	GM	Range	MBIC_50_	MBIC_90_	GM
*Cercozoa*	32–128	32	128	50.8	64–256	64	256	101.6	64–256	64	256	101.6
*Charophyta*	64–4096	512	1024	542.4	64–4096	512	1024	588.1	64–4096	512	1024	530.1
*Chlorophyta*	8–8192	512	2048	469.2	8–8192	512	2048	472.8	8–8192	256	1024	291.1
*Cryptophyta*	32–2048	128	2048	203.2	32–2048	128	2048	203.2	32–2048	128	2048	203.2
Cyanobacteria	16–4096	256	2048	297	16–4096	256	10,248	339.2	16–4096	256	2048	255.3
*Euglenophyta*	128–512	256	512	256	128–512	256	512	256	128–512	256	512	256
*Glaucophyta*	128–2048	512	2048	512	128–2048	512	2048	512	128–2048	512	2048	512
*Haptophyta*	32–1024	128	512	120.8	32–1024	256	1024	170.9	32–1024	256	1024	170.9
*Miozoa*	32–2048	128	1024	194	64–2048	128	1024	222.9	16–2048	128	1024	154
*Ochrophyta*	128–1024	256	1024	298.6	128–1024	256	1024	314.4	128–1024	256	512	249.5
*Rhodophyta*	64–128	128	1024	181	64–128	128	1024	181	64–128	128	1024	181
Unknown	16–256	128	256	101.6	32–256	128	256	114	2–128	64	128	45.3

**Table 3:** Antibiofilm activity against *Candida* spp. of marine natural products. Range: range of concentrations tested; MBIC_50_: lowest concentration of the test compound that resulted in ≥50% inhibition of biofilm formation; MBIC_90_: lowest concentration of the test compound that resulted in ≥90% inhibition of biofilm formation; GM: geometric mean.

Phylum	*Candida albicans*				*Candida parapsilosis*			
	Range	MBIC_50_	MBIC_90_	GM	Range	MBIC_50_	MBIC_90_	GM
*Cercozoa*	32–64	32	64	40.3	32–128	32	128	80.8
*Charophyta*	8–128	16	64	23.7	32–4096	256	512	284
*Chlorophyta*	2–2048	32	256	44.7	8–4096	256	1024	224.6
*Cryptophyta*	4–128	8	128	12.7	16–1024	64	1024	101.6
Cyanobacteria	2–2048	32	256	39.6	16–4096	256	1024	186.6
*Euglenophyta*	8–16	8	16	12.7	64–256	128	256	128
*Glaucophyta*	4–256	8	256	16	64–1024	256	1024	256
*Haptophyta*	0.5–256	32	128	20.2	16–512	128	512	90.5
*Miozoa*	8–512	32	256	44.2	32–1024	64	1024	122.2
*Ochrophyta*	4–256	32	256	27.4	64–1024	128	512	165.5
*Rhodophyta*	8–64	16	64	20.2	32–512	64	512	90.5
Unknown	16–64	32	64	28.5	16–256	64	256	64

**Table 4:** Biofilm values obtained by crystal violet elution of each microorganism analyzed in this study. OD_580_ (optical density at 580 nm).

Microorganism Strains	OD_580_	Type
*Escherichia coli*	1.552	Bacteria
*Klebsiella pneumoniae*	0.934	Bacteria
*Enterobacter cloacae*	0.879	Bacteria
*Pseudomonas aeruginosa*	0.701	Bacteria
*Staphylococcus aureus*	0.918	Bacteria
*Staphylococcus epidermidis*	1.129	Bacteria
*Staphylococcus hominis*	0.940	Bacteria
*Candida albicans*	1.014	Yeast
*Candida parapsilosis*	1.301	Yeast

## Supplementary Materials

The following are available online at https://www.mdpi.com/2079-6382/8/2/77/s1. Table S1: Three methods of extraction analyzed by one-way ANOVA. DF: degrees of freedom; P value: *p* < 0.05 were considered statistically significant. Table S2: Results of Gram-negatives strains comparing with treatment (phylum) and method of extraction (solvent). Two-way ANOVA followed by *post hoc* Tukey’s test were used. MD: mean difference; SD: standard error of the difference; CI of diff: confidence interval of difference 95%; P value: *p* < 0.05 were considered statistically significant. Table S3: Results of Gram-positives strains comparing with treatment (phylum) and method of extraction (solvent). Two-way ANOVA followed by *post hoc* Tukey’s test were used. MD: mean difference; SD: standard error of the difference; CI of diff: confidence interval of difference 95%; P value: *p* < 0.05 were considered statistically significant. Table S4: Results of Candida spp. comparing with treatment (phylum) and method of extraction (solvent). Two-way ANOVA followed by post hoc Tukey’s test were used. MD: mean difference; SD: standard error of the difference; CI of diff: confidence interval of difference 95%; P value: *p* < 0.05 were considered statistically significant.
