# Supplementary material for: Inhibition of Bacterial and Fungal Biofilm Formation by 675 Extracts from Microalgae and Cyanobacteria
Source: Antibiotics (Basel). 2019 Jun 12;8(2):77. doi: 10.3390/antibiotics8020077 (PMC6628188; doi:10.3390/antibiotics8020077)
Supplement: Supplementary file 1 [file antibiotics-08-00077-s001.pdf]

antibiotics

Supplementary Materials

Table S1. Three methods of extraction analyzed by one-way ANOVA. DF: degrees of freedom; P value: p <0.05 were considered statistically significant.

Source of variance	Sum of squares	DF	Mean of squares	P value
Treatment	0.3933	2	0.1966	0.3272
Residual	1065	6051	0.1760	
Total	1065	6053		

Table S2. Results of Gram-negative strains comparing with treatment (phylum) and method of extraction (solvent). Two-way ANOVA followed by *post hoc* Tukey's test were used. MD: mean difference; SD: standard error of the difference; CI of diff: confidence interval of difference 95%; P value: p <0.05 were considered statistically significant.

Escherichia coli				
	MD	SD	CI of diff.	P value
Charophyta vs *Chlorophyta*	-0.1043	0.0192	-0.1495 to -0.0591	<0.0001[3]
Charophyta vs Cyanobacteria	-0.0641	0.0194	-0.1096 to -0.0186	0.0028[1]
Chlorophyta vs Cyanobacteria	0.0402	0.0118	0.0124 to 0.0680	0.0021[1]
Klebsiella pneumoniae				
	MD	SD	CI of diff.	P value
Charophyta vs *Chlorophyta*	-0.0433	0.0231	-0.0976 to 0.0110	0.1473[4]
Charophyta vs Cyanobacteria	0.0010	0.0232	-0.0536 to 0.0554	0.9992[4]
Chlorophyta vs Cyanobacteria	0.0442	0.0141	0.0112 to 0.0772	0.0050[1]
Enterobacter cloacae				
	MD	SD	CI of diff.	P value
Charophyta vs *Chlorophyta*	-0.01127	0.02871	-0.0787 to 0.0562	0.9186[4]
Charophyta vs Cyanobacteria	0.02747	0.02880	-0.0402 to 0.0951	0.6065[4]
Chlorophyta vs Cyanobacteria	0.03873	0.01746	-0.0023 to 0.0798	0.0690[4]
Pseudomonas aeruginosa				
	MD	SD	CI of diff.	P value
Charophyta vs *Chlorophyta*	-0.08730	0.02849	-0.1542 to -0.0204	0.0064[1]
Charophyta vs Cyanobacteria	-0.05450	0.02866	-0.1218 to 0.0129	0.1391[4]
Chlorophyta vs Cyanobacteria	0.03280	0.01746	-0.0082 to 0.0739	0.1460[4]

Tukey's *post hoc* test significance; [1]$p < 0.01$; [2]$p < 0.001$; [3]$p < 0.0001$; [4]ns.

Table S3. Results of Gram-positive strains comparing with treatment (phylum) and method of extraction (solvent). Two-way ANOVA followed by *post hoc* Tukey's test were used. MD: mean difference; SD: standard error of the difference; CI of diff: confidence interval of difference 95%; P value: p <0.05 were considered statistically significant.

Staphylococcus aureus				
	MD	SD	CI of diff.	P value
Charophyta vs *Chlorophyta*	-0.3021	0.04860	-0.4163 to -0.1879	<0.0001[3]
Charophyta vs Cyanobacteria	-0.05780	0.04875	-0.1724 to 0.0568	0.4622[4]
Chlorophyta vs Cyanobacteria	0.2443	0.02956	0.1748 to 0.3137	<0.0001[3]
Staphylococcus epidermidis				
	MD	SD	CI of diff.	P value
Charophyta vs *Chlorophyta*	-0.2050	0.06332	-0.3538 to -0.0562	0.0036[1]
Charophyta vs Cyanobacteria	-0.08300	0.06351	-0.2322 to 0.0662	0.3919[4]
Chlorophyta vs Cyanobacteria	0.1220	0.03851	0.03151 to 0.2125	0.0046[1]
Staphylococcus hominis				
	MD	SD	CI of diff.	P value
Charophyta vs *Chlorophyta*	-0.1177	0.05585	-0.2489 to 0.0135	0.0892[4]
Charophyta vs Cyanobacteria	0.2093	0.05602	0.0777 to 0.3410	0.0006[2]
Chlorophyta vs Cyanobacteria	0.3270	0.03397	0.2472 to 0.4068	<0.0001[3]

Tukey’s *post hoc* test significance; [1]p < 0.01; [2]p < 0.001; [3]p < 0.0001; [4]ns.

Table A4. Results of Candida spp. comparing with treatment (phylum) and method of extraction (solvent). Two-way ANOVA followed by *post hoc* Tukey's test were used. MD: mean difference; SD: standard error of the difference; CI of diff: confidence interval of difference 95%; P value: p <0.05 were considered statistically significant.

Candida albicans				
	MD	SD	CI of diff.	P value
Charophyta vs *Chlorophyta*	0.0049	0.0226	-0.04818 to 0.0580	0.9744[2]
Charophyta vs Cyanobacteria	-0.0266	0.0227	-0.07981 to 0.0267	0.4701[2]
Chlorophyta vs Cyanobacteria	-0.0315	0.0135	-0.06375 to 0.0008	0.0579[2]
Candida parapsilopsis				
	MD	SD	CI of diff.	P value
Charophyta vs *Chlorophyta*	-0.136	0.0281	-0.2017 to -0.0698	<0.0001[1]
Charophyta vs Cyanobacteria	-0.0603	0.0282	-0.1265 to 0.0058	0.0824[2]
Chlorophyta vs Cyanobacteria	0.0754	0.0171	0.03524 to 0.1155	<0.0001[1]

Tukey’s *post hoc* test significance; [1]p < 0.0001; [2]ns.
